# Prognosis and transition of multi-site pain during the course of 5 years: Results of knee pain and function from a prospective cohort study among 756 adolescents

**DOI:** 10.1371/journal.pone.0250415

**Published:** 2021-05-21

**Authors:** Sinead Holden, Ewa M. Roos, Christian Lund Straszek, Jens Lykkegaard Olesen, Martin Bach Jensen, Thomas Graven-Nielsen, Michael Skovdal Rathleff

**Affiliations:** 1 Center for General Practice at Aalborg University, Aalborg, Denmark; 2 Department of Health Science and Technology, Aalborg University, Aalborg, Denmark; 3 Research Unit for Musculoskeletal Function and Physiotherapy, Institute of Sports Science and Clinical Biomechanics, University of Southern Denmark, Odense, Denmark; 4 Institute of Sports Medicine Copenhagen, Copenhagen University Hospital, Bispebjerg, Denmark; 5 Department of Health Science and Technology, Center for Neuroplasticity and Pain (CNAP), SMI, Aalborg University, Aalborg, Denmark; Monash University, AUSTRALIA

## Abstract

**Introduction:**

Multi-site pain has not been investigated among adolescents suffering from knee pain. This study aimed to examine the trajectory of pain in adolescents with knee-pain, to determine if multi-site pain in adolescents together with other established prognostic factors (frequency of pain, sex, sports participation, Health Related Quality of Life (HRQoL)) was associated with five-year prognosis of knee-pain and function.

**Methods:**

This prospective cohort study included 504 adolescents with knee pain and 252 controls. At five-year follow-up, participants responded to an questionnaire which documented prescence and severity of knee pain and co-occurring pain.

**Results:**

At follow-up, 358 (71.0%) of those with knee-pain at baseline, and 182 (72.2%) controls responded. Female sex, low HRQoL, daily pain, and multi-site pain were associated with an increased odds of knee pain after 5 years (odds ratio: 1.41–3.37). Baseline multi-site pain was not associated with problems running at follow-up, whereas higher sports participation at baseline was associated with less problems running at follow-up (odd ratio 0.49). Among those with knee-pain at inclusion, the number of pain sites increased from a median of 2 (IQR 1–3) to 4 (IQR 2–6) at follow-up (P<0.05). Those with multi-site pain at follow-up score significantly worse in self-reported knee function, compared to those with one pain site only.

**Conclusion:**

This study identified a set of factors that appeared to be associated with an increased risk of knee pain at five years follow up. Research is needed to understand and help direct treatment of adolescents with multi-site pain.

## Introduction

Up to one third of adolescents regularly experience musculoskeletal pain, with back and knee pain being the most common [1, 2]. Musculoskeletal pain negatively affects health-related quality of life (HRQoL), physical activity and function, and is associated with anxiety, depression and sleep problems [3–6]. In the United States, estimated directs costs of pain in adolescents are 19.6 billion dollars annually [7]. Adolescent pain can be highly persistent, and is associated with a higher risk of suffering from pain during adulthood [4, 8, 9]. Pain during adolescence is also associated with mental health disorders, social welfare benefits and work absenteeism in early adulthood [10, 11] highlighting the impact for the individual and burden to society.

Adolescent knee pain has a high propensity towards chronicity with nearly one in two reporting knee pain after two and five years with continued impact [12]. This seems to contrast with adolescent back pain, which is typically characterized by short transient periods of pain in adolescents [13] with only a subset experiencing a persistent burden of back pain into early adulthood [14]. Understanding prognostic factors will be critical in facilitating targeted treatment for adolescents with the highest risk of a poor longer-term prognosis [15].

Previous research on adults has shown that pain in more than one location may be a strong and easily captured prognostic factor across multiple pain conditions/locations [16, 17]. A recent study evaluated the patterns of pain in adolescents, and found that 1 in 5 classified as having ‘multi-site bodily pain’ (primarily presenting in the back, knee, shoulder and head) which was associated with low HRQoL, female sex and lower sports participation [18], compared to those with localised pain only. Despite knee pain is the most common single location, adolescents with knee pain often experience pain in more than one location [2, 18–20] with a tendency for an increasing number of pain sites over time [19]. This has been suggested to be partially as a result of facilitated pain mechanisms [21], and was recently identified as a characteristic of youth with long standing knee pain since adolescence [22].

This underscores the need to examine the progression of pain, and role of multi-site pain in adolescent knee pain. Data focusing on this critical development period of the life span are limited [15], with multi-site pain not evaluated as a potential prognostic factor in a recent systematic review [23].

The aim of this analysis was to investigate the trajectories of pain, and the role of multi-site pain as a prognostic factor for pain and function (evaluated as self-reported problems running), together with common health and activity related factors for persistent knee pain in a prospective cohort of adolescents. We aimed to evaluate if multi-site pain is prognostic after controlling for sex and pain frequency (common prognostic factors in MSK pain), and if health related quality of life and sports activity have further independent association with prognosis of pain.

## Materials and methods

### Study design and setting

The study was approved by the local ethics committee of North Denmark Region (Den Videnskabsetiske Komité for Region Nordjylland; N-20110020). The ethics committee did not require an individually signed consent form, but did require that the schools informed the parents about the study and that participation in the study was voluntary. This analysis was conducted using data from a prospective population-based cohort study [24]. The primary aim of the cohort was to quantify the persistence and impact of adolescent knee pain, and was pre-registered on clinical trials (NCT02873143). A formal sample size calculation was not undertaken, as a sample of convenience was used, and exploring prognostic factors were not the main aim of the follow-up. This paper therefore represents an exploratory analysis.

### Study population and recruitment

The cohort (APA 2011 cohort) was recruited from upper secondary schools in Aalborg in 2011. This included a total of 2846 potential responders, of whom 2200 aged 15–19 responded to the questionnaire, and were included in the baseline questionnaire. From this, adolescents reporting at least monthly or more frequent knee pain were invited to participate in the prospective cohort (670 adolescents). Of these 670, 60 of the adolescents did not report telephone numbers. From the 610 with contact details, 504 indicated at least monthly knee pain and were included in this prospective cohort study. In addition, 252 controls who reported no knee pain were also randomly selected to participate as the control group. All 756 were also included in the 2-year follow-up, conducted in 2013 [3].

### Exposures

The baseline questionnaire contained demographic information (age, sex, height, weight) and a mannequin with labelled body parts (on both front and back), where participants reported if there was a specific body region where they had pain. If they had pain, they were asked about frequency of pain in the selected regions: rarely, monthly, weekly, more than once per week, or almost daily. Participants also answered questions about participation in leisure-time sport and, if yes, how many times per week and the Knee Injury and Osteoarthritis Outcome Score; KOOS [25]). Finally, HRQoL was measured using the EuroQoL 5-Dimensions (EQ-5D 3L).

### Follow-up

All 504 adolescents with knee pain, and 252 controls, were contacted in September 2016 to participate in the 5-year follow-up answer an online questionnaire (link sent via email). The questionnaire included basic demographic information, on current height and weight.

The prescence of knee pain at five year follow-up was pre-defined as the primary outcome. Specifically, all participants (both those with a history of knee pain as adolescents and controls without knee pain as adolescents) were asked if they had knee pain in the previous week which was considered the primary outcome. If participants reported ‘yes’ to experiencing knee pain in the previous week, they were asked how often they experience pain. In addition to this, participants indicated whether they had experienced pain in the previous month in any of the 11 predefined locations they were asked at baseline. They also reported on other health-related outcomes including function (Knee Injury Osteoarthritis Outcome Score; KOOS [25]). Participants were offered a cinema ticket after they responded to the questionnaire.

### Statistical analysis

The analysis of prognostic factors was done using a hierarchical logistic regression (i.e. hierarchical general linear model with a binomial distribution and logit link function).

The primary outcome was knee-pain at follow-up, defined by participants answering ‘Yes’ to the question ‘Have you had knee pain in the previous week’. Block one included the following baseline exposures; sex, pain frequency and duration of pain. Block two included the baseline exposure of multi-site pain to evaluate its impact on the model beyond these characteristics. The final block included quality of life and sports participation to determine if they further explained the variance in outcome.

Due to the potential limitations in missed or incorrect cases based on the definition of pain in the previous week, a second sensitivity analysis was done based on whether participants *reported a frequency of weekly pain* at a minimum (i.e. weekly, several times per week, or daily pain in response to ‘how often do you feel your knee pain’) and answered ‘Yes’ to pain in the previous week *or* month.

Finally, a separate model was run for problems running, as a functional outcome. This was evaluated by question 2 on the ‘function sport and activities’ subscale of the KOOS ‘degree or difficulty running. This was dichotomised into moderate to severe problems running (Yes/No).

Chi squared tests were used to examine differences in proportions of pain at other locations, between those with continuing longstanding knee pain since baseline (persistent knee pain), those with knee pain at baseline reporting no knee pain in the past week (recovered), and those with no pain at baseline (control). For significance, adjusted standardised residuals (>2) were used. An adjusted standard residual of >2 indicates a greater observed cell value than would be expected if no association was present.

One-way analysis of variance was used to detect for differences in patient reported outcomes (KOOS knee function) between those with and without multi-site pain at follow-up. The dependent variables were the five KOOS domains (pain, symptoms, sport and recreation, quality of life and activities of daily living) while the independent variable was the binary variable of multi-site pain, categorised based on if participants reported pain in >1 location at follow-up.

## Results

At five-year follow-up, 358 adolescents (71.0% of the original cohort) with knee pain and 182 (72.2%) from the control group responded (Table 1). Of these, 40.5% (95%CI: 35.4–45.6%) of adolescents with knee pain at baseline reported knee pain 5 years later compared to 13.2% (95%CI: 8.2–18.1) from the control group who developed knee pain since baseline.

**Table 1:** Baseline demographics.

	Knee pain in 2011 (n = 504)	No knee pain in 2011 (n = 226)
Age [years]*	17 (17–18)	17 (16–18)
**Sex % females (95% CI)**	72.0 (67.9–75.8)	62 (55.5–68.0)
**Weight [kg] (SD)**	65.2 (11.5)	64.0 (12.8)
**Height [cm] (SD)**	172.1 (9.2)	172.7 (8.8)
**BMI (SD)**	22.0 (3.1)	21.4 (3.5)
**% who participate in leisure-time sports (95% CI)**	71.5 (67.3–75.3)	66.4 (55.3–77.4)
Sport sessions per week* **(all participants)**	2 (0–4)	3 (2–4)
EQ-5D index*	0.78 (0.72–0.82)	1 (0.82–1)
EQ-5D-vas*	75 (60–85)	90 (80–95)
Average pain duration (months)*	24 (12–42)	N/A
**% with a non-traumatic onset (95% CI)**	68.3 (64.0–72.2)	N/A
**Pain frequency (95%CI)**		N/A
** Daily (%)**	27.5 (23.7–31.6)	N/A
** Several times per week**	19.0 (15.8–22.3)	N/A
** Weekly**	28.9 (25.1–33.1)	N/A
** Monthly**	24.0 (20.5–28.0)	N/A

* Median and interquartile range.

BMI: Body mass index.

EQ-5D: EuroQol 5 Dimensions.

### Transitions of knee pain at baseline, two and five years

**Fig 1:**
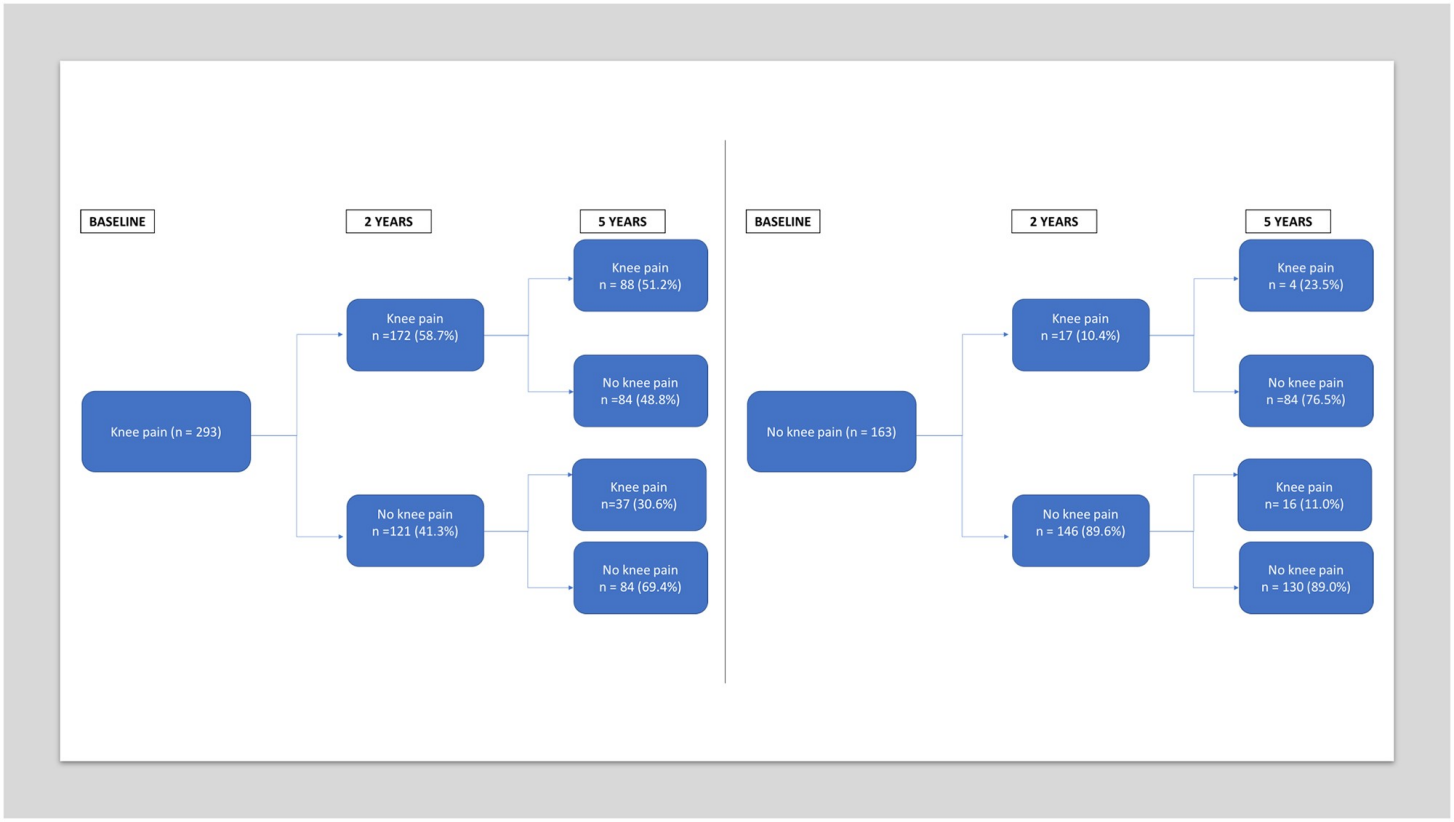
Pain transitions at two and five years for those with data available on knee pain at baseline (N = 253) and control (N = 163).

The pain transitions across showed that a higher proportion of participants reporting pain at both baseline and two-year follow-up also reported pain at five-years, compared to controls or those reporting pain at baseline only (Fig 1).

### Pain in other locations

Overall, there was a significant increase in number of pain-sites from baseline to five year follow-up (P<0.05). Participants with knee pain at baseline reported a median of 2 (IQR 1–3) pain sites, which increased to 4 (IQR 2–5) at follow-up.

**Fig 2:**
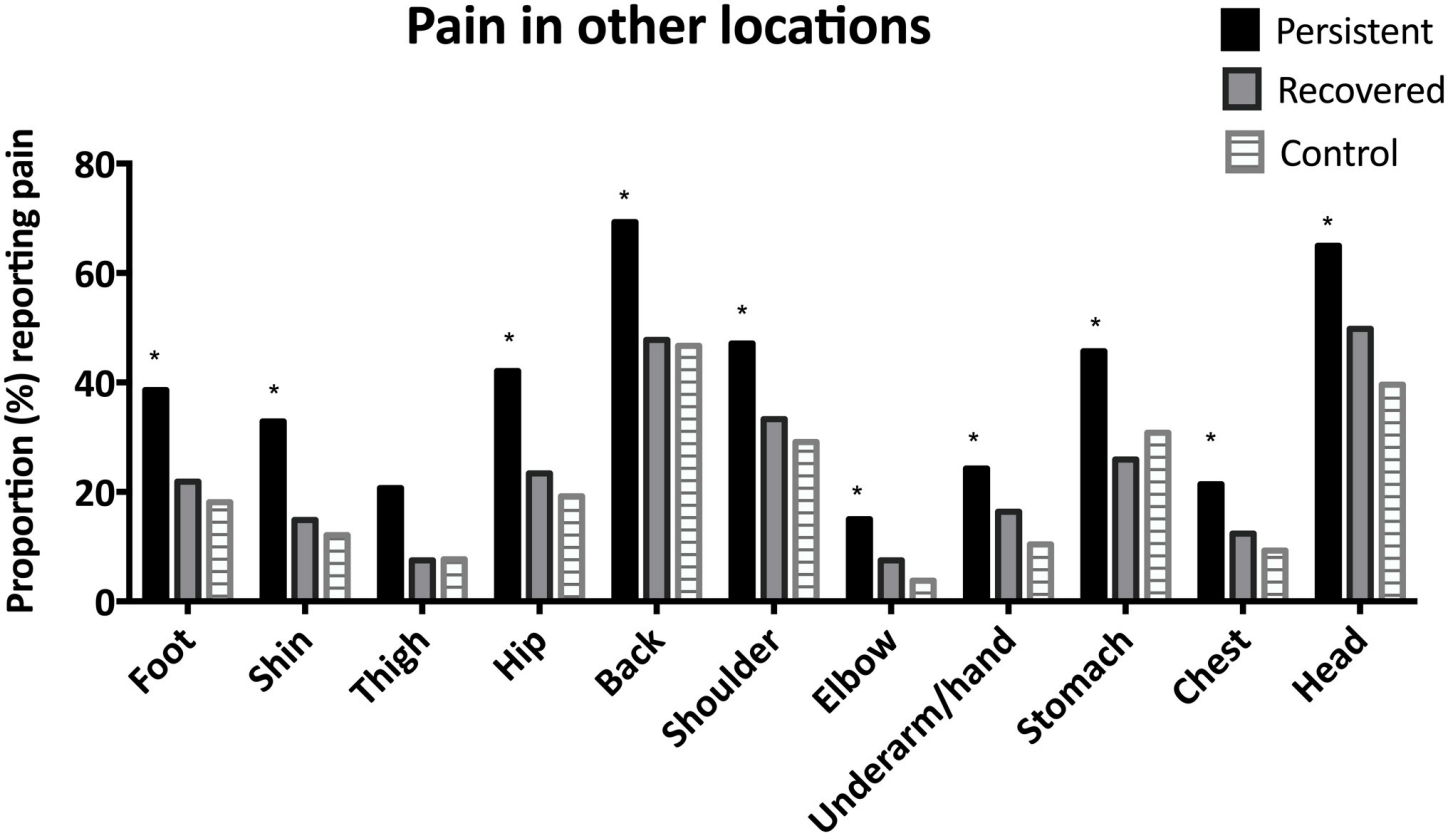
Percentage of those with persistent knee pain, recovered from adolescent knee pain, and controls reporting pain in other locations at 5-year follow-up. * adjusted standardised residuals indicates a significant deviance from expected proportion.

At five-year follow-up there was significant asscociation between group (persistent knee pain *versus* recovered *versus* control) and the proportion of participants reporting pain at other locations (shoulder ***χ***^**2**^ = 11.9; p <0.005), underarm/hand χ2 = 11.0;p<0.005, thigh χ2 = 18.0; p<0.001, hip χ2 = 23.2; p<0.001, foot χ2 = 19.3; p<0.001, elbow χ2 = 13.3; p<0.005, shin χ2 = 25.5; p<0.001, chest χ2 = 10.2; p<0.05, back χ2 = 20.0; p< 0.001, head χ2 = 20.5; p<0.001, and stomach χ2 = 15.3; p< 0.001). Those with persistent knee pain had a significantly greater pain than expected in all other locations (Fig 2) (indicated by adjusted standardised residuals > 2). Those with pain in more than one location at follow-up scored significantly worse on all KOOS domains compared to those with pain in onle location only at follow-up (Table 2).

**Table 2:** Comparison of KOOS scores in those with single versus multi-site pain at follow-up.

	Single site pain at five-year follow-up	Multi-site pain at five year follow-up
KOOS Pain	90 (87–93)	84 (82–86)^#^
KOOS Symptoms	62 (60–65)	59 (58–60)^#^
KOOS ADL	95 (93–97)	91 (89–92) ^#^
KOOS Sport and Recreation	82 (78–87)	71 (68–74) ^#^
KOOS QoL	65 (63–67)	62 (60–63)*

*P <0.05

^#^ P <0.01.

ADL: activities of daily living; QoL: Quality of Life.

### Prognostic factors for knee pain at follow-up

For the outcome of knee pain in the previous week, the first Model (Block1; sex, pain frequency and duration) was statistically significant (Chi Square (5) = 33.4; p<0.0005). Sex (p<0.0005) and daily pain (p<0.001) were statistically significant predictors in the model, but pain duration did not add statistically significantly to the model (p = 0.438). Being female was the strongest predictor in Block 1, and increased the odds of having knee pain at follow up by 3.37 (95%CI 1.91 to 5.92).

The addition of multi-site pain in Block 2 significantly improved model performance (Step Chi Square = 3.682; df = 1; P<0.05), with multi-site pain increasing odds of knee pain by 1.635; 95%CI 1.0 to 2.68; P = 0.052).

The final model was statistically significant (Chi-Square = 54.079; df 11; p<0.0005). The addition of quality of life and sports participation significantly improved the model (Step Chi squared (5) = 14.02; p = 0.015). Compared to those in the 75^th^ to 100^th^ percentile of EQ 5D scores, those in the lowest quartile had 4 times (1.42 to 11.93; p<0.01) increased odds of having knee pain. Baseline sports participation was not significant (p>0.05) in the final block.

### Sensitivity analyses

The final model for the sensitivity analysis for using a stricter definition of pain (at least weekly pain) was statistically significant, with similar odds of the predictor variables and minor changes in estimates for most variables (S2 File). Unsurprisingly, as the definition of pain for the sensitivity analysis was based on a threshold for frequency, baseline pain frequency was most strongly associated in this model, with those having daily pain having nearly three times the odds of reporting weekly pain at follow-up (online web Appendix A of S1 File).

One hundred and thirteen participants reported moderate to severe problems running at follow-up (with 27 of them reporting no knee pain). Pain duration and sports participation at baseline were associated with a lower risk of reporting problems running after 5 years (online web-Appendix B of S1 File). In contrast to the models for knee pain, HRQoL and multi-site pain were not associated with problems running at follow-up.

## Discussion

### Principle findings

This five year cohort study showed that in adoelscents with knee pain, female sex, lack of sports participation, low HRQoL, and high frequency of pain are associated with an increased risk of reporting knee pain in early adulthood. Importantly, these factors were also associated with self reported difficulties running. This is particularly important as a previous systematic review concluded a lack of prognostic studies for functional outcomes in musculoskeletal pain conditions.

For prescence of knee pain at follow-up, multi-site pain at baseline significantly improved the model over and above other known prognostic characteristics- sex, pain frequency and duration. Number of pain sites appear to increase over time which may indicate spreading of pain complaints and those continuing to have knee pain at follow-up had the highest proportions of pain in other locations (including non-MSK locations such as stomach/head), with back pain being the most common. Those with pain in multiple locations scored significantly worse in all domains of the KOOS outcomes. This study is an important contribution into prognosis of MSK pain during the critical developmental period transitioning from adolescence into early adulthood, which is under-researched in the area of musculoskeletal pain [15].

### Prognosis and multi-site pain

Pain in additional sites/locations has been identified as a prognostic factor for MSK pain, across a range of different pain locations in adults [16, 17]. Research among adolescent populations has shown that those with more pain locations are more likely to experience continued persistent pain [19, 20, 26]. In this study, the odds ratio of 1.65 indicates multi-site pain may be important, but HRQoL and female sex were more strongly associated with prognosis. Interestingly multi-site pain was only associated with pain as an outcome, and not function. This underscores that ‘pain predicts pain’ but being able to participate in sport as an adolescent is a more important determinant of future functional problems in early adulthood.

### Progression of pain over time

Previous cohort studies have identified stable patterns of number of pain sites in adults [27, 28], giving support to the idea that certain pain trajectories may develop early in life, and persist once established. This aligns with trajectories of low back pain transitioning from adolescence into adulthood, where 60% display consistently either low or high prevalence of back pain and its’ impact, while a smaller proportion display a trajectory of either improving (15%) or worsening (22%) of symptoms [14].

In this cohort, adolescents with persistent knee pain reported more pain at all pain-sites at follow-up. This corresponds with research showing that pain in one location, is an independent risk factor for developing pain in subsequent other pain-free locations [29]. Similarly, in adolescents, lower extremetiy pain has been linked to the development of subsequent back pain complaints [30]. There are several potential mechanisms which may underpin this association. Sensitisation of central pain mechanisms are thought to play a role [31], with data suggesting a link between how widespread pain is, and pain sensitivity [32]. Our previous data on younger adolescents with longstanding patellofemoral pain (a non-specific pain complaint common in adoelscents), highlighted that higher temporal summation of pain at baseline was associated with poorer improvements in pain during 12-weeks treatment [33]. Holley et al. [34] also demonstrated that decreased pain modulation predicted those who transitioned to persistent pain in youth with new onset musculoskeletal pain. Another explanation could be the fact that knee pain is often due to over-use/ physical activity related, which could inherently contain risks of other musculoskeletal injuries/pain complaints. At this stage, it is not possible to determine if the association is mechanistically associated with the presence of long standing pain itself, or due to other potential shared risk factors/ mechanisms.

### Implications

Pain in more than one location may be associated with prognosis of pain, and seems to progress as a significantly proportion of the young adults with persistent knee pain at follow-up reported pain in other locations, which increased from baseline. Understanding and decreasing the burden of adolescent pain may in time help decrease the burden of pain during adulthood [15]. Emerging evidence indicates that a specific set of factors may increase an individual’s risk of a poor prognosis [23]. Identifying the profiles of adolescents at high risk for poor prognosis will be important to guide appropriate distribution of health care resources towards those who need it most, at a time which the negative trajectory is most susceptible to change [35, 36]. Increased sports participation at baseline was more associated with a favourable prognosis for functional limitations. This raises the question as whether advising adolescents with knee pain to remain active, while being careful not to aggravate symptoms would increase long-term running ability.

### Limitations

By relying on the self-report of symptoms of sites where adolescents experienced pain, it is possible that we captured some relatively mild additional pain complaints in the analysis of multi-site pain. We may therefore underestimate the relationship between multi-site pain and prognosis seen in clinically relevant pain complaints originating from clinical practice and not a population-based sample as this. Similarly, at follow-up we did not undertake a clinical exam for knee pain, or pain in other locations and cannot determine if pain is due to actual or potential tissue damage or if they were acute or longstanding pain. A further limitation is that at follow-up we did not capture a severity measure of the additional pain locations, which may be partially offset by the use of a control group from the same background population. We did not adjust for other factors when exploring the association of pain in other locations at follow-up.

## Conclusion

This study identified a set of factors, including female sex, worse quality of life and multi-site pain, that were associated with an increased risk of knee pain at five years follow up. Additionally, baseline sports participation was associated with less problems running at follow-up. At follow-up those with pain had significantly more pain in all other locations than controls or those who recovered from adolescent knee pain. Importantly, this is the first study to evaluate these factors during the critical transition into adulthood which may determine future health outcomes. Future research should try and determine how best to use prognostic profiles to inform optimal use of health-care resources in this critical period of life.

## Supporting information

S1 File (DOCX)

S2 File (PDF)
