# Supplementary material for: Prognosis and transition of multi-site pain during the course of 5 years: Results of knee pain and function from a prospective cohort study among 756 adolescents
Source: PLoS One. 2021 May 21;16(5):e0250415. doi: 10.1371/journal.pone.0250415 (PMC8139498; doi:10.1371/journal.pone.0250415)
Supplement: S1 File — (DOCX) [file pone.0250415.s001.docx]

Appendix A: More strict pain definition (pain in past week or month if minimum weekly pain)

| **Variable (Baseline values)** | **Odds Ratio** | **95% CI for odds ratio** | **P-value** |
| --- | --- | --- | --- |
| **Block 1** |  |  |  |
| Sex (female compared to male) | 2.93 | 1.60- 5.35 | <0.0005 |
| Pain duration (per 10 month increase) | 1.00 | 1.00- 1.01 | 0.366 |
| Pain frequency (compared to monthly knee pain) |  |  |  |
| Weekly | 1.93 | 0.94- 3.99 | 0.075 |
| Several times per week | 2.31 | 1.07- 5.01 | 0.030 |
| Daily | 4.14 | 2.05 – 8.33 | <0.001 |
| **Block 2** |  |  |  |
| Sex | 2.85 | 1.56- 5.3 | 0.001 |
| Pain duration (per 10 month increase) | 1.00 | 1.0- 1.014 | 0.177 |
| Pain frequency (compared to monthly knee pain) |  |  |  |
| Weekly | 1.90 | 0.92- 3.93 | 0.084 |
| Several times per week | 2.31 | 1.07- 5.01 | 0.034 |
| Daily | 4.18 | 2.07- 8.5 | <0.0005 |
| Multi-site pain (compared to no multisite pain) | 1.43 | 0.85 to 2.40 | 0.18 |
| **Block 3** |  |  |  |
| Sex | 2.58 | 1.38 – 4.79 | 0.003 |
| Pain duration (per 10 month increase) | 1.00 | 0.99 – 1.01 | 0.644 |
| Pain frequency (compared to monthly knee pain) |  |  |  |
| Weekly | 1.72 | 0.81 – 3.62 | 0.156 |
| Several times per week | 1.82 | 0.8 – 4.13 | 0.154 |
| Daily | 2.85 | 1.31 – 6.12 | 0.008 |
| Multi- site pain | 1.41 | 0.83 – 2.39 | 0.210 |
|  |  |  |  |
| Participation in sports (compared with no leisure time sport) |  |  |  |
| 1-2 times per week | 0.68 | 0.35 – 1.31 | 0.243 |
| 3 or more times per week | 0.75 | 0.42- 1.34 | 0.336 |
| EQ- 5D index score (compared with 75- 100% percentile) |  |  |  |
| 50-75% percentile | 2.12 | 0.72 – 6.32 | 0.174 |
| 25-50% percentile | 1.96 | 0.66 – 5.78 | 0.223 |
| 0-25% percentile | 3.41 | 1.16 – 10.06 | 0.02 |

Appendix B: Poor function as outcome (defined as moderate or severe problems running).

| **Variable (Baseline values)** | **Odds Ratio** | **95% CI for odds ratio** | **P- value** |
| --- | --- | --- | --- |
| **Block 1** |  |  |  |
| Sex | 2.26 | 1.25 - 4.12 | 0.007 |
| Pain duration (per 10 month increase) | 1.01 | 1.00 - 1.02 | 0.114 |
| Pain frequency (compared to monthly knee pain) |  |  |  |
| Weekly | 1.91 | 0.91 - 3.99 | 0.087 |
| Several times per week | 2.13 | 0.96 - 4.70 | 0.062 |
| Daily | 3.93 | 1.93 - 7.99 | <0.0005 |
| **Block 2** |  |  |  |
| Sex | 2.27 | 1.25 - 4.13 | 0.007 |
| Pain duration (per 10 month increase) | 1.01 | 1.00 - 1.02 | 0.113 |
| Pain frequency (compared to monthly knee pain) |  |  |  |
| Weekly | 1.91 | 0.91 - 4.00 | 0.087 |
| Several times per week | 2.13 | 0.96 - 4.70 | 0.062 |
| Daily | 3.93 | 1.93 - 7.99 | <0.0005 |
| Multi- site pain | 0.97 | 0.58 - 1.62 | 0.898 |
| **Block 3** |  |  |  |
| Sex | 2.01 | 1.09 - 3.70 | 0.026 |
| Pain duration (per 10 month increase) | 1.01 | 1.00 - 1.02 | 0.349 |
| Pain frequency (compared to monthly knee pain) |  |  |  |
| Weekly | 1.68 | 0.78 - 3.58 | 0.183 |
| Several times per week | 1.73 | 0.75 - 3.98 | 0.197 |
| Daily | 2.84 | 1.30 - 6.20 | 0.009 |
| Multi- site pain | 0.96 | 0.57 - 1.63 | 0.883 |
|  |  |  |  |
| Participation in sports (compared with no leisure time sport) |  |  |  |
| 1- 2 times per week | 0.78 | 0.41 - 1.47 | 0.440 |
| 3 or more times per week | 0.49 | 0.27 - 0.88 | 0.016 |
| EQ- 5D index score (compared with 75- 100% percentile) |  |  |  |
| 50- 75% percentile | 1.11 | 0.41 - 3.05 | 0.832 |
| 25- 50% percentile | 1.66 | 0.63 - 4.39 | 0.309 |
| 0- 25% percentile | 1.79 | 0.67 - 4.82 | 0.249 |
