# Supplementary material for: Prognosis and transition of multi-site pain during the course of 5 years: Results of knee pain and function from a prospective cohort study among 756 adolescents
Source: PLoS One. 2021 May 21;16(5):e0250415. doi: 10.1371/journal.pone.0250415 (PMC8139498; doi:10.1371/journal.pone.0250415)
Supplement: S2 File — (PDF) [file pone.0250415.s002.pdf]

5 års opfølgning

* Required

Anvender du en smartphone? Så hold den som anvist på billedet imens du udfylder spørgeskemaet.

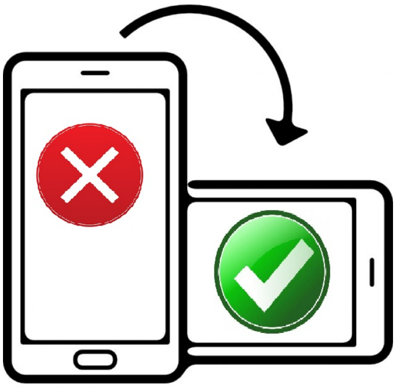

1. **Navn (fornavn og efternavn)** *

2. **E-mail (Skriv den e-mail adresse som dette spørgeskema blev sendt til).** *

3. **Telefonnummer** *

4. **Alder** *

5. **Vægt (kg)** *

6. **Højde (cm)** *

7. **Har du haft knæsmerter inden for den sidste uge?** *
   Mark only one oval.
   - Ja
   - Nej

8. **Har du haft knæsmerter i løbet af den sidste måned?** *
Mark only one oval.

- Ja
- Nej *After the last question in this section, skip to question 10.*

9. **Såfremt du ikke længere har knæsmerter, hvornår ophørte de?**
Mark only one oval.

- Indenfor de sidste 3 måneder
- Indenfor de sidste 6 måneder
- Indenfor de sidste 12 måneder
- Indenfor de sidste 2 år
- Jeg har fortsat knæsmerter
- Jeg har aldrig haft knæsmerter

10. **Hvor ofte mærker du knæsmerter?** *
Mark only one oval.

- Næsten dagligt
- Flere gange om ugen
- Ugentligt
- Månedligt
- Sjældent
- Aldrig *Skip to question 16.*

11. **Har dine knæsmerter indflydelse på:** *
Marker de svar som passer til Din situation
Check all that apply.

- [ ] Dit aktivitetsniveau
- [ ] Dit humør
- [ ] Din hverdag
- [ ] Knæsmerterne har ikke haft indflydelse på nogle af de ovenstående
- [ ] Other: ....

12. **Hvor mange gange om ugen dyrker du sport? (f.eks. løb, gå i fitnesscenter, fodbold eller lignende)** *
Mark only one oval.

- 0 gange om ugen
- 1 gang om ugen
- 2 gange om ugen
- 3 gange om ugen
- 4 gange om ugen
- 5 gange om ugen
- 6 gange om ugen
- 7 gange om ugen
- Mere end 7 gange om ugen

13. **Har du reduceret din deltagelse i sport eller stoppet helt med at dyrke en sport på grund af knæsmerter?** *
Mark only one oval.

- Ja
- Nej

14. **Har du skiftet sportsgren på grund af knæsmerter?** *
Mark only one oval.

- Ja
- Nej *Skip to question 16.*

15. **Noter din(e) tidligere samt din(e) nuværende sportsgren(e)** *

....

....

....

....

16. **Har du svært ved at falde i søvn om aftenen?** *
Mark only one oval.

- Nej, overhovedet ikke
- Ja, nogle aftener
- Ja, de fleste aftener

17. **Vågner du flere gange om natten?** *
Mark only one oval.

- Nej, overhovedet ikke
- Ja, nogle nætter
- Ja, de fleste nætter

18. **Har du svært ved at sove igennem om natten?** *

Mark only one oval.

- ( ) Nej, overhovedet ikke
- ( ) Ja, nogle nætter
- ( ) Ja, de fleste nætter

19. **Føler du dig træt om morgenen når du vågner?** *

Mark only one oval.

- ( ) Nej, overhovedet ikke
- ( ) Ja, nogen morgener
- ( ) Ja, de fleste morgener

20. **Har du modtaget behandling for knæsmerter i løbet af de seneste 12 måneder?** *

Check all that apply.

- [ ] Nej, jeg har ikke modtaget behandling for knæsmerter
- [ ] Ja, hos en ortopædkirurg
- [ ] Ja, hos en kiropraktor
- [ ] Ja, hos en fysioterapeut
- [ ] Ja, hos en reumatolog
- [ ] Ja, hos min praktiserende læge
- [ ] Other: ....

21. **Hvilken slags behandling har du modtaget?** *

Check all that apply.

- [ ] Træning (f.eks. Styrketræning, træning med elastik og lignende)
- [ ] Smertestillende medicin
- [ ] Indlægssåler (Specialfremstillede skosåler)
- [ ] Massage
- [ ] Akupunktur
- [ ] Jeg har ikke modtaget nogen behandling for knæsmerter
- [ ] Other: ....

22. **Tager du smertestillende medicin?** *

Mark only one oval.

- ( ) Ja
- ( ) Nej *Skip to question 25.*

23. **Hvilken type smertestillende medicin tager du?**

....

....

....

....

24. **Hvor ofte tager du smertestillende medicin?** *

Mark only one oval.

- Aldrig
- En gang om måneden
- En gang om ugen
- En gang om dagen
- Flere gange om dagen

25. **Har du i de sidste 30 dage oplevet smerter i en eller flere af de følgende regioner?** *

Mark only one oval per row.

	Venstre	Højre	Begge sider	Ingen smerter
Skulder				
Underarm/hånd				
Lår				
Hofte/lysken				
Fod				
Albue				
Skinneben				
Knæ				

26. **Har du i de sidste 30 dage oplevet smerter i en eller flere af de følgende regioner?** *

Mark only one oval per row.

	Ja	Nej
Bryst		
Ryg		
Hoved		
Mave		

27. **Har dine knæsmerter haft indflydelse på dit valg af uddannelse eller job?** *

Mark only one oval.

- Ja
- Nej *Skip to question 29.*

28. **Marker de områder hvor dine knæsmerter har haft indflydelse på dit arbejdsliv.**
Check all that apply.

- [ ] Valg af uddannelse
- [ ] Valg af job

29. **Hvad er den værste smerte i knæet du har oplevet den seneste uge?** *
Mark only one oval.

	0	1	2	3	4	5	6	7	8	9	10	
Ingen smerte	⬭	⬭	⬭	⬭	⬭	⬭	⬭	⬭	⬭	⬭	⬭	Værst tænkelig smerte

KOOS

Vejledning:
Dette spørgeskema indeholder spørgsmål om, hvordan Du oplever dit knæ. Informationerne vil hjælpe os til at følge med i hvordan Du har det og hvor godt Du klarer dig i dagligdagen. Ved hvert spørgsmål skal Du sætte et kryds i det alternativ, der passer bedst på dig. Du må kun sætte ét kryds ved hvert spørgsmål. Hvis Du er i tvivl om hvad Du skal svare, skal Du sætte krydset ved det alternativ, der føles mest rigtigt.

Symptom

Tænk på de knægener Du har haft i løbet af den sidste uge, når Du besvarer de næste spørgsmål.

30. **S1 Har knæet været hævet** *
Mark only one oval per row.

	Aldrig	Sjældent	Ind imellem	Ofte	Altid
	⬭	⬭	⬭	⬭	⬭

31. **S2 Har Du haft murren i knæet, hørt klik eller andre lyde fra knæet, når Du bevæger det ?** *
Mark only one oval per row.

	Aldrig	Sjældent	Ind imellem	Ofte	Altid
	⬭	⬭	⬭	⬭	⬭

32. **S3 Har knæet haget sig fast eller været låst ?** *
Mark only one oval per row.

	Aldrig	Sjældent	Ind imellem	Ofte	Altid
	⬭	⬭	⬭	⬭	⬭

33. **S4 Kan Du strække knæet helt ?** *
Mark only one oval per row.

	Altid	Ofte	Ind imellem	Sjældent	Aldrig
	⬭	⬭	⬭	⬭	⬭

34. **S5 Kan Du bøje knæet helt ?** *

Mark only one oval per row.

	Altid	Ofte	Ind imellem	Sjældent	Aldrig
	⬭	⬭	⬭	⬭	⬭

Stivhed

Følgende spørgsmål handler om ledstivhed. Ledstivhed medfører besvær med at komme igang eller øget modstand, når Du bøjer eller strækker knæet. Angiv i hvor høj grad Du har oplevet ledstivhed i knæet i løbet af den sidste uge.

35. **S6 Hvor stift er dit knæ, når Du lige er vågnet om morgenen ?** *

Mark only one oval per row.

	Slet ikke	Lidt	Moderat	Meget	Ekstremt
	⬭	⬭	⬭	⬭	⬭

36. **S7 Hvor stift er dit knæ senere på dagen, når Du har siddet eller ligget og hvilet ?** *

Mark only one oval per row.

	Slet ikke	Lidt	Moderat	Meget	Ekstremt
	⬭	⬭	⬭	⬭	⬭

Smerte

37. **P1 Hvor ofte har Du ondt i knæet ?** *

Mark only one oval per row.

	Aldrig	Hver måned	Hver uge	Hver dag	Altid
	⬭	⬭	⬭	⬭	⬭

Hvor mange knæsmerter har Du haft i løbet af den sidste uge, under følgende aktiviteter ?

38. **P2 Dreje/vride på belastet knæ** *

Mark only one oval per row.

	Ingen	Lette	Moderate	Stærke	Ekstremt
	⬭	⬭	⬭	⬭	⬭

39. **P3 Strække knæet helt** *

Mark only one oval per row.

	Ingen	Lette	Moderate	Stærke	Ekstremt
	⬭	⬭	⬭	⬭	⬭

40. **P4 Bøje knæet helt** *

Mark only one oval per row.

	Ingen	Lette	Moderate	Stærke	Ekstremt
	◯	◯	◯	◯	◯

41. **P5 Gå på jævnt underlag** *

Mark only one oval per row.

	Ingen	Lette	Moderate	Stærke	Ekstremt
	◯	◯	◯	◯	◯

42. **P6 Gå op eller ned ad trapper** *

Mark only one oval per row.

	Ingen	Lette	Moderate	Stærke	Ekstremt
	◯	◯	◯	◯	◯

43. **P7 Om natten (smerter, som forstyrrer din søvn)** *

Mark only one oval per row.

	Ingen	Lette	Moderate	Stærke	Ekstremt
	◯	◯	◯	◯	◯

44. **P8 Siddende eller liggende** *

Mark only one oval per row.

	Ingen	Lette	Moderate	Stærke	Ekstremt
	◯	◯	◯	◯	◯

45. **P9 Stående** *

Mark only one oval per row.

	Ingen	Lette	Moderate	Stærke	Ekstremt
	◯	◯	◯	◯	◯

Funktion i dagligdagen

Følgende spørgsmål omhandler dit fysiske formåen. Angiv hvilken grad af besvær Du har oplevet under følgende aktiviteter i løbet af den sidste uge, på grund af dine knæproblemer.

46. **A1 Gå ned ad trapper** *

Mark only one oval per row.

	Intet	Lidt	Moderat	Stort	Ekstremt
	◯	◯	◯	◯	◯

47. **A2 Gå op ad trapper** *

Mark only one oval per row.

	Intet	Lidt	Moderat	Stort	Ekstremt
	⬭	⬭	⬭	⬭	⬭

48. **A3 Rejse dig fra siddende** *

Mark only one oval per row.

	Intet	Lidt	Moderat	Stort	Ekstremt
	⬭	⬭	⬭	⬭	⬭

Angiv graden af besvær Du har oplevet ved hver aktivitet i løbet af den sidste uge

49. **A4 Stå stille** *

Mark only one oval per row.

	Intet	Lidt	Moderat	Stort	Ekstremt
	⬭	⬭	⬭	⬭	⬭

50. **A5 Gå ned i knæ, f.eks. for at samle noget op fra gulvet** *

Mark only one oval per row.

	Intet	Lidt	Moderat	Stort	Ekstremt
	⬭	⬭	⬭	⬭	⬭

51. **A6 Gå på jævnt underlag** *

Mark only one oval per row.

	Intet	Lidt	Moderat	Stort	Ekstremt
	⬭	⬭	⬭	⬭	⬭

52. **A7 Gå ind/ud af en bil** *

Mark only one oval per row.

	Intet	Lidt	Moderat	Stort	Ekstremt
	⬭	⬭	⬭	⬭	⬭

53. **A8 Tage på indkøb** *

Mark only one oval per row.

	Intet	Lidt	Moderat	Stort	Ekstremt
	⬭	⬭	⬭	⬭	⬭

54. **A9 Tage strømper på** *

Mark only one oval per row.

	Intet	Lidt	Moderat	Stort	Ekstremt
	⬭	⬭	⬭	⬭	⬭

55. **A10 Stå ud af sengen** *

Mark only one oval per row.

	Intet	Lidt	Moderat	Stort	Ekstremt

56. **A11 Tage strømper af** *

Mark only one oval per row.

	Intet	Lidt	Moderat	Stort	Ekstremt

57. **A12 Ligge i sengen (vende dig, have knæet i samme stilling i lang tid)** *

Mark only one oval per row.

	Intet	Lidt	Moderat	Stort	Ekstremt

58. **A13 Stige ind og ud af badekar/brusebad** *

Mark only one oval per row.

	Intet	Lidt	Moderat	Stort	Ekstremt

59. **A14 Sidde** *

Mark only one oval per row.

	Intet	Lidt	Moderat	Stort	Ekstremt

60. **A15 Sætte dig og rejse dig fra toilettet** *

Mark only one oval per row.

	Intet	Lidt	Moderat	Stort	Ekstremt

61. **A16 Udføre tungt husarbejde (vaske gulv, støvsuge, bære øl/sodavandskasser o.lign.)** *

Mark only one oval per row.

	Intet	Lidt	Moderat	Stort	Ekstremt

62. **A17 Udføre let husarbejde (lave mad, tørre støv af etc.)** *

Mark only one oval per row.

	Intet	Lidt	Moderat	Stort	Ekstremt

Funktion, sport og fritid

Følgende spørgsmål handler om din fysiske formåen. Angiv hvilken grad af besvær Du har oplevet under følgende aktiviteter i løbet af den sidste uge på grund af dine knæproblemer.

63. **SP1 Sidde i hug** *

Mark only one oval per row.

	Intet	Lidt	Moderat	Stort	Ekstremt

64. **SP2 Løbe** *

Mark only one oval per row.

	Intet	Lidt	Moderat	Stort	Ekstremt

65. **SP3 Hoppe** *

Mark only one oval per row.

	Intet	Lidt	Moderat	Stort	Ekstremt

66. **SP4 Dreje/vride på belastet knæ** *

Mark only one oval per row.

	Intet	Lidt	Moderat	Stort	Ekstremt

67. **SP5 Ligge på knæ** *

Mark only one oval per row.

	Intet	Lidt	Moderat	Stort	Ekstremt

Livskvalitet

68. **Q1 Hvor ofte bliver Du mindet om dit knæproblem ?** *

Mark only one oval per row.

	Aldrig	Hver måned	Hver uge	Hver dag	Altid

69. **Q2 Har Du forandret din måde at leve på for at undgå at overbelaste knæet ?** *

Mark only one oval per row.

	Slet ikke	Noget	Moderat	I stor udstrækning	Totalt

70. **Q3 I hvor stor grad kan Du stole på dit knæ ?** *
Mark only one oval per row.

	Fuldt ud	I stor udstrækning	Moderat	Til en vis grad	Slet ikke
	⬭	⬭	⬭	⬭	⬭

71. **Q4 Hvor store problemer har Du almindeligvis med dit knæ ?** *
Mark only one oval per row.

	Ingen	Små	Moderat	Store	Ekstreme
	⬭	⬭	⬭	⬭	⬭

EQ-5D

Angiv, ved at sætte kryds i én af kasserne i hver gruppe, hvilke udsagn, der bedst beskriver din helbredstilstand i dag

72. **Bevægelighed** *
Mark only one oval.

- ⬭ Jeg har ingen problemer med at gå omkring
- ⬭ Jeg har nogle problemer med at gå omkring
- ⬭ Jeg er bundet til sengen

73. **Personlig pleje** *
Mark only one oval.

- ⬭ Jeg har ingen problemer med min personlige pleje
- ⬭ Jeg har nogle problemer med at vaske mig eller klæde mig på
- ⬭ Jeg kan ikke vaske mig eller klæde mig på

74. **Sædvanlige aktiviteter (fx.arbejde, studie, husarbejde, familie- eller fritidsaktiviteter)** *
Mark only one oval.

- ⬭ Jeg har ingen problemer med at udføre mine sædvanlige aktiviteter
- ⬭ Jeg har nogle problemer med at udføre mine sædvanlige aktiviteter
- ⬭ Jeg kan ikke udføre mine sædvanlige aktiviteter

75. **Smerter/ubehag** *
Mark only one oval.

- ⬭ Jeg har ingen smerter eller ubehag
- ⬭ Jeg har moderate smerter eller ubehag
- ⬭ Jeg har ekstreme smerter eller ubehag

76. **Angst/depression** *
Mark only one oval.

- ⬭ Jeg er ikke ængstelig eller deprimeret
- ⬭ Jeg er moderat ængstelig eller deprimeret
- ⬭ Jeg er ekstremt ængstelig eller deprimeret

IPAQ

Vi ønsker at undersøge de forskellige former for fysisk aktivitet, mennesker udfører i dagligdagen. Spørgsmålene vedrører den tid, du i løbet af de sidste 7 dage har tilbragt med fysisk aktivitet. Du bedes besvare hvert spørgsmål, selvom du selv ikke synes, at du er en aktiv person. Angiv venligst aktiviteter på dit arbejde, aktiviteter som udgør en del af arbejdet hjemme eller i haven, aktiviteter for at komme fra et sted til et andet, og i fritiden aktiviteter i forbindelse med afslapning, motion eller idræt.

Angiv alle meget anstrengende aktiviteter, som du udførte de sidste 7 dage. Ved meget anstrengende fysisk aktivitet forstås aktiviteter, der kræver hård fysisk anstrengelse, som får dig til at trække vejret meget mere end normalt. Angiv kun fysiske aktiviteter, som du har udført i mindst 10 minutter ad gangen.

77. **1. Hvor mange dage har du de sidste 7 dage udført meget anstrengende fysiske aktiviteter som løftet tunge ting, gravet, aerobics eller cyklet hurtigt?** *

Mark only one oval.

- 1 dag
- 2 dage
- 3 dage
- 4 dage
- 5 dage
- 6 dage
- 7 dage
- Ingen meget anstrengende aktiviteter *Skip to question 79.*

78. **2. Hvor meget tid tilbringer du som regel med meget anstrengende fysiske aktiviteter på en af disse dage?**

Mark only one oval.

- 10 minutter pr. dag
- 20 minutter pr. dag
- 30 minutter pr. dag
- 40 minutter pr. dag
- 50 minutter pr. dag
- 1 time pr. dag
- 2 timer pr. dag
- 3 timer pr. dag
- 4 timer pr. dag
- over 4 timer pr. dag
- Ved ikke/ikker sikker

Angiv alle moderate aktiviteter, som du udførte de sidste 7 dage. Ved moderat fysisk aktivitet forstås aktiviteter, der kræver moderat fysisk anstrengelse, som får dig til at trække vejret noget mere end normalt. Angiv kun fysiske aktiviteter, som du har udført i mindst 10 minutter ad gangen.

79. **3. Hvor mange dage har du de sidste 7 dage udført moderate fysiske aktiviteter som båret lette genstande, cyklet i regelmæssigt tempo eller spillet tennisdouble? Medregn ikke gåture.** *
Mark only one oval.

- 1 dag
- 2 dage
- 3 dage
- 4 dage
- 5 dage
- 6 dage
- 7 dage
- Ingen moderate aktiviteter *Skip to question 81.*

80. **4. Hvor meget tid tilbringer du som regel med moderate fysiske aktiviteter på en af disse dage?**
Mark only one oval.

- 10 minutter pr. dag
- 20 minutter pr. dag
- 30 minutter pr. dag
- 40 minutter pr. dag
- 50 minutter pr. dag
- 1 time pr. dag
- 2 timer pr. dag
- 3 timer pr. dag
- 4 timer pr. dag
- over 4 timer pr. dag
- Ved ikke/ikke sikker

Angiv den tid de sidste 7 dage, du tilbragte med gåture. Dette er inklusive gåture på arbejdet eller hjemme, gåture i forbindelse med rejser fra et sted til et andet, og alle andre gåture kun i forbindelse med afslapning, idræt, motion eller fritid.

81. **5. Hvor mange dage har du de sidste 7 dage gået mindst 10 minutter ad gangen?** *
Mark only one oval.

- 1 dag
- 2 dage
- 3 dage
- 4 dage
- 5 dage
- 6 dage
- 7 dage
- Ingen gåture (Gå til spørgsmål 7) *Skip to question 83.*

82. **6. Hvor meget tid tilbringer du som regel med gåture på en af disse dage?**

Mark only one oval.

- 10 minutter pr. dag
- 20 minutter pr. dag
- 30 minutter pr. dag
- 40 minutter pr. dag
- 50 minutter pr. dag
- 1 time pr. dag
- 2 timer pr. dag
- 3 timer pr. dag
- 4 timer pr. dag
- over 4 timer pr. dag
- Ved ikke/ikke sikker

Det sidste spørgsmål vedrører den tid, du har brugt på at sidde ned på ugedage de sidste 7 dage. Medregn tid tilbragt på arbejdet, hjemme, mens du laver kursusarbejde og i fritiden. Dette kan være inklusive den tid, du har tilbragt ved et skrivebord, med venner, når du har læst eller har siddet eller lagt dig for at se TV.

83. **7. Hvor meget tid de sidste 7 dage har du brugt på at sidde ned på en ugedag?**

Mark only one oval.

- Under 1 time pr. dag
- 1 time pr. dag
- 2 timer pr. dag
- 3 timer pr. dag
- 4 timer pr. dag
- over 4 timer pr. dag
- Ved ikke/ikke sikker

Du er nu færdig med at besvare spørgeskemaet. Tak for din deltagelse.

Powered by Google Forms
